# Trends in Binge Drinking and Heavy Alcohol Consumption Among Pregnant Women in the US, 2011 to 2020

**DOI:** 10.1001/jamanetworkopen.2022.24846

**Published:** 2022-08-01

**Authors:** Jeffrey T. Howard, Jessica K. Perrotte, Kassandra Flores, Caleb Leong, Joseph David Nocito, Krista J. Howard

**Affiliations:** 1 College for Health, Community and Policy, Department of Public Health, University of Texas at San Antonio; 2 Department of Psychology, Texas State University, San Marcos

## Abstract

This cross-sectional study compares trends in the prevalence of binge drinking and heavy alcohol consumption among pregnant and nonpregnant women from 2011 through 2020.

## Introduction

Alcohol-related mortality has been increasing among women in the US during the past 2 decades.^1^ Although no recent studies have found increased alcohol-related mortality specifically among pregnant women, 1 study^2^ found increases in combined drug- and alcohol-related mortality. Recent data on binge drinking among pregnant women suggests a modest increase from 2011 to 2018^3^ and no increase from 2018 to 2020.^4^ However, little is known about how longer trends in problematic alcohol use may differ between pregnant and nonpregnant women. We sought to compare trends in prevalence of binge drinking and heavy alcohol consumption among pregnant and nonpregnant women from 2011 through 2020.

## Methods

This cross-sectional study obtained public use data from the Behavioral Risk Factor Surveillance System (BRFSS) from January 1, 2011, to December 31, 2020. The BRFSS is a nationally representative, cross-sectional sample of US adults that measures alcohol consumption based on 30-day recall. The BRFSS defines binge drinking as 4 or more drinks during a single occasion and heavy alcohol consumption as 8 or more drinks per week (eMethods and eTable in the Supplement). Pregnancy status (pregnant vs not pregnant) was the primary exposure; sex was self-reported by all participants in the BRFSS data set, and all pregnant participants identified as women. Race and ethnicity (Hispanic or Latino, non-Hispanic Black, non-Hispanic White, non-Hispanic multiple races or ethnicities, and non-Hispanic other race or ethnicity) are reported in the BRFSS, and the distribution differs significantly by the primary exposure (pregnancy), which should be accounted for when comparing prevalence between pregnant and nonpregnant women. Analyses were adjusted for age group (18-24, 25-29, 30-34, 35-39, and 40-44 years) and race and ethnicity. The University of Texas at San Antonio Institutional Review Board waived the need for approval because the research did not involve human participants. This study followed the STROBE reporting guideline.

Age- and race and ethnicity–adjusted prevalence of binge drinking and heavy alcohol consumption were estimated using logistic regression models adjusted for complex survey design and weighting. Log linear regression models were used to estimate average annual percentage change (AAPC) in prevalence rates with 95% CIs. Two-sided *P* < .05 was considered statistically significant. Data were analyzed using R, version 4.0.2 (R Foundation for Statistical Computing).

## Results

Among the 49 098 pregnant women included in the study, 28.8% were 18 to 24 years of age and 3.7% were 40 to 44 years of age compared with 26.2% and 19.1%, respectively, for the 1 243 402 nonpregnant women (*P* < .001). Pregnant women were less likely to be non-Hispanic White (50.5%) compared with nonpregnant women (54.4%) (*P* < .001) (Table). The prevalence of binge drinking increased from 2.5% (95% CI, 1.6%-3.4%) in 2011 to 6.1% (95% CI, 2.2%-10.0%) in 2020 for pregnant women, an AAPC of 8.9% (95% CI, 4.8%-12.9%; *P* = .003) (Figure). Binge drinking for nonpregnant women decreased from 18.6% (95% CI, 17.8%-19.3%) in 2011 to 17.6% (95% CI, 16.8%-18.5%) in 2020 with an AAPC of 0.7% (95% CI, −0.5% to 1.8%; *P* = .28), reflecting an increase from 2012 to 2019. Prevalence of heavy alcohol consumption increased from 0.7% (95% CI, 0.3%-1.0%) in 2011 to 3.2% (95% CI, 0.6%-5.8%) in 2020 for pregnant women, an AAPC of 11.6% (95% CI, 4.0%-19.3%; *P* = .02). Prevalence of heavy alcohol consumption for nonpregnant women increased from 6.6% (95% CI, 6.1%-7.1%) in 2011 to 7.5% (95% CI, 6.9%-8.1%) in 2020, an AAPC of 2.3% (95% CI, 0.9%-3.7%; *P* = .01).

**Table.** Descriptive Statistics of Study Characteristics for Binge Drinking and Heavy Alcohol Consumption Samples, Behavioral Risk Factor Surveillance System, 2011 Through 2020

Variable	Study sample					
	Binge drinking (n = 646 504)			Heavy alcohol consumption (n = 645 996)		
	Group, No. (weighted %)^a^		*P* value^b^	Group, No. (weighted %)^a^		*P* value^b^
	Pregnant	Not pregnant		Pregnant	Not pregnant	
Year						
2011	2967 (9.1)	72 224 (9.6)	.02	2967 (9.1)	72 159 (9.6)	.03
2012	2827 (9.9)	71 019 (10.1)		2823 (9.9)	70 952 (10.1)	
2013	2967 (11.0)	70 976 (10.0)		2959 (11.0)	70 895 (10.0)	
2014	2428 (9.4)	62 370 (9.8)		2427 (9.4)	62 364 (9.8)	
2015	2343 (10.5)	59 164 (10.1)		2341 (10.5)	59 113 (10.1)	
2016	2343 (10.5)	59 164 (10.1)		2341 (10.5)	59 113 (10.1)	
2017	2404 (10.7)	58 813 (10.1)		2398 (10.7)	58 721 (10.1)	
2018	2287 (10.0)	58 200 (10.2)		2281 (10.0)	58 050 (10.2)	
2019	2005 (9.6)	54 426 (9.9)		2003 (9.6)	54 446 (9.9)	
2020	1991 (9.3)	55 586 (10.0)		1996 (9.3)	55 647 (10.1)	
Age, y						
18-24	5444 (28.8)	105 900 (26.2)	<.001	5435 (28.8)	105 658 (26.2)	<.001
25-29	6792 (26.9)	94 917 (16.3)		6785 (26.9)	94 808 (16.3)	
30-34	7253 (27.6)	118 257 (19.6)		7248 (27.6)	118 159 (19.6)	
35-39	3810 (12.5)	133 630 (17.0)		3801 (12.4)	133 574 (17.0)	
40-44	1110 (3.7)	146 154 (19.1)		1111 (3.7)	146 226 (19.1)	
Missing	153 (0.5)	23 084 (1.9)		156 (0.5)	23 0356 (1.9)	
Race and ethnicity						
Hispanic or Latino	4154 (26.1)	88 120 (21.1)	<.001	4148 (26.1)	87 934 (21.0)	<.001
Non-Hispanic						
Black	2169 (12.8)	62 723 (13.4)		2167 (12.8)	62 616 (13.4)	
White	15 412 (50.5)	404 446 (54.4)		15 402 (50.5)	404 364 (54.4)	
Multiple races	671 (1.6)	17 221 (1.8)		668 (1.6)	17 180 (1.8)	
Other^c^	1853 (7.8)	40 443 (8.0)		1847 (7.8)	40 409 (8.0)	
Missing	303 (1.3)	8989 (1.4)		304 (1.3)	8957 (1.4)	
Alcohol consumption						
Binge drinking	754 (3.8)	108 483 (17.8)	<.001		NA	<.001	
Heavy	NA	NA		306 (1.4)	38 651 (6.3)	

Abbreviation: NA, not applicable.

^a^ Counts are unweighted; percentages reflect adjustment for complex sample design and population weighting.

^b^ Based on Rao-Scott χ^2^ statistic.

^c^ Includes American Indian or Alaska Native, Asian, and Native Hawaiian or other Pacific Islander.

**Figure.**
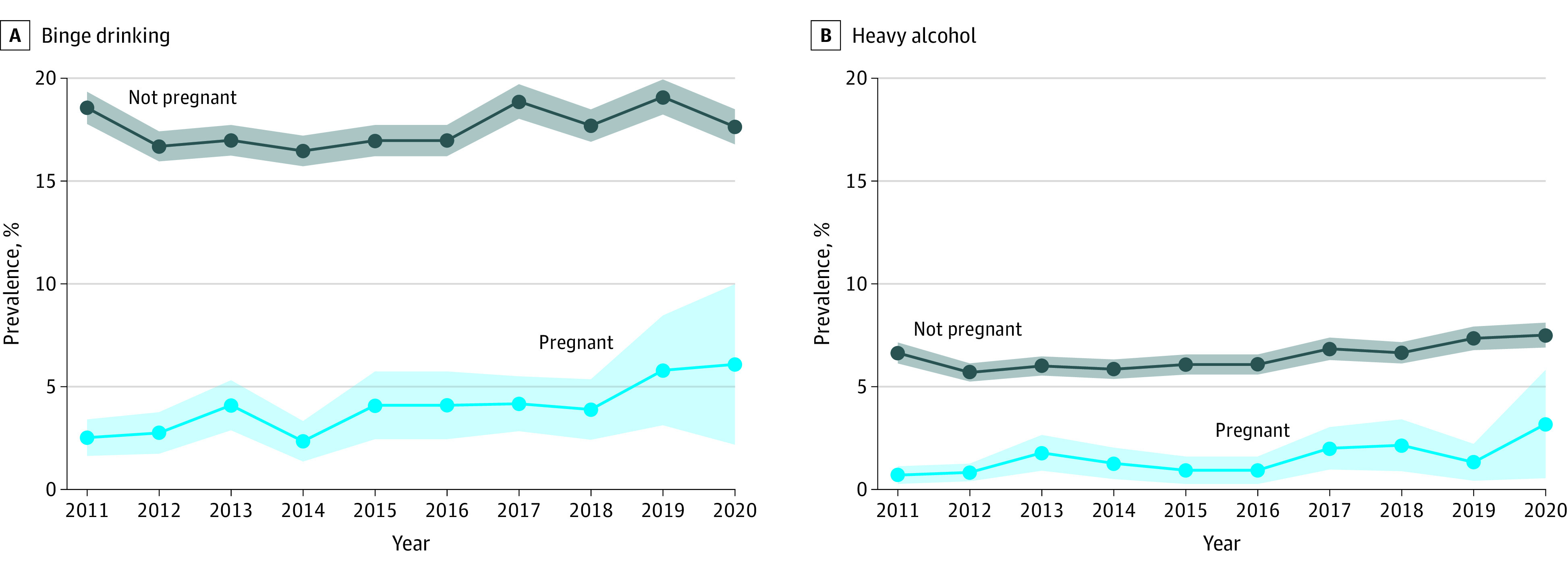
Prevalence of Binge Drinking and Heavy Alcohol Consumption Among Pregnant and Nonpregnant Women, 2011 to 2020 Data are from the public use Behavioral Risk Factor Surveillance System. A, Estimated average annual percent change for binge drinking was 8.9% (95% CI, 4.8%-12.9%; *P* = .003) among pregnant women and 0.7% (95% CI, −0.5% to 1.8%; *P* = .28) among nonpregnant women. B, Estimated average annual percent change for heavy alcohol consumption was 11.6% (95% CI, 4.0%-19.3%; *P* = .02) among pregnant women and 2.3% (95% CI, 0.9%-3.7%; *P* = .01) among nonpregnant women.

## Discussion

In this cross-sectional study, we found that binge drinking and heavy alcohol consumption were higher among nonpregnant women than pregnant women, but the AAPC for both behaviors was significantly greater among pregnant women than nonpregnant women. Binge drinking increased by 0.7% per year between 2012 and 2019, and heavy alcohol consumption increased by 2.3% per year among nonpregnant women. However, binge drinking increased 8.9% per year and heavy alcohol consumption increased 11.6% per year among pregnant women.

Study limitations include the cross-sectional design, self-reported alcohol consumption, and wide CIs for estimates in 2019 and 2020. These results suggest worsening behavioral risks among pregnant women, potentially owing to changes in socioeconomic^5^ and psychosocial stressors^6^ that may have been exacerbated by the COVID-19 pandemic.

## Notes

Supplement. **eTable.** Alcohol Consumption Questions in the Behavioral Risk Factor Surveillance System (BRFSS) **eMethods.** Use of Behavioral Risk Factor Surveillance System Data
eReferences
