# Supplementary material for: Trends in Binge Drinking and Heavy Alcohol Consumption Among Pregnant Women in the US, 2011 to 2020
Source: JAMA Netw Open. 2022 Aug 1;5(8):e2224846. doi: 10.1001/jamanetworkopen.2022.24846 (PMC9344359; doi:10.1001/jamanetworkopen.2022.24846)
Supplement: Supplement. — eTable. Alcohol Consumption Questions in the Behavioral Risk Factor Surveillance System (BRFSS) eMethods. Use of Behavioral Risk Factor Surveillance System Data eReferences [file jamanetwopen-e2224846-s001.pdf]

Supplementary Online Content

Howard JT, Perrotte JK, Flores K, Leong C, Nocito JD III, Howard KJ. Trends in binge drinking and heavy alcohol consumption among pregnant women in the US, 2011 to 2020. *JAMA Netw Open*. 2022;5(8):e2224846. doi:10.1001/jamanetworkopen.2022.24846

eTable. Alcohol Consumption Questions in the Behavioral Risk Factor Surveillance System (BRFSS)
eMethods. Use of Behavioral Risk Factor Surveillance System Data
eReferences

This supplementary material has been provided by the authors to give readers additional information about their work.

© 2022 Howard JT et al. *JAMA Network Open*.

eTable. Alcohol Consumption Questions in the Behavioral Risk Factor Surveillance System (BRFSS)

Question
1. During the past 30 days, how many days per week or per month did you have at least one drink of any alcoholic beverage such as beer, wine, a malt beverage or liquor?
2. One drink is equivalent to a 12-ounce beer, a 5-ounce glass of wine, or a drink with one shot of liquor. During the past 30 days, on the days when you drank, about how many drinks did you drink on the average?
3. Considering all types of alcoholic beverages, how many times during the past 30 days did you have X [CATI X = 5 for men, X = 4 for women] or more drinks on an occasion?
4. During the past 30 days, what is the largest number of drinks you had on any occasion?

eMethods. Use of Behavioral Risk Factor Surveillance System Data

The Behavioral Risk Factor Surveillance System (BRFSS) uses the 4 questions in eTable 1 to measure the frequency and number of alcoholic beverages reported in the past 30 days by each respondent[1]. Based on the responses to these questions, two risky drinking patterns are then calculated from the responses[2], including binge drinking and heavy alcohol consumption. For women, if 1 <= question 3 <=76, then they are considered a binge drinker. Heavy alcohol consumption is derived from the combination of questions 1 and 2. First, question 1 is divided by 7 days per week or 30 days per month, depending on whether the respondent reported days per week or month, then the result is multiplied by the answer to question 2 to yield the average number of drinks per week. For women, if the average number of drinks per week > 7 then the respondent is coded as a heavy drinker. Both binge drinking and heavy alcohol consumption were included in this analysis because they are considered excessive drinking patterns that are associated with other health risks, including mortality[3].

© 2022 Howard JT et al. *JAMA Network Open*.

eReferences

1. Center for Disease Control and Prevention. 2020 BRFSS Questionnaire. Centers for Disease Control and Prevention. https://www.cdc.gov/brfss/questionnaires/pdf-ques/2020-BRFSS-Questionnaire-508.pdf. Published 2021. Accessed 6/14/2022.
2. Center for Disease Control and Prevention. Calculated Variables in the 2020 Data File of the Behavioral Risk Factor Surveillance System. Centers for Disease Control and Prevention. https://www.cdc.gov/brfss/annual_data/2020/pdf/2020-calculated-variables-version4-508.pdf. Published 2021. Accessed 6/14/2022.
3. Xi B, Veeranki SP, Zhao M, Ma C, Yan Y, Mi J. Relationship of Alcohol Consumption to All-Cause, Cardiovascular, and Cancer-Related Mortality in U.S. Adults. *Journal of the American College of Cardiology.* 2017;70(8):913-922.

© 2022 Howard JT et al. *JAMA Network Open*.
